# Evaluating clinical characteristics studies produced early in the Covid-19 pandemic: A systematic review

**DOI:** 10.1371/journal.pone.0251250

**Published:** 2021-05-18

**Authors:** Lakshmi Manoharan, Jonathan W. S. Cattrall, Carlyn Harris, Katherine Newell, Blake Thomson, Mark G. Pritchard, Peter G. Bannister, Louise Sigfrid, Tom Solomon, Peter W. Horby, Gail Carson, Piero Olliaro

**Affiliations:** 1 International Severe Acute Respiratory and Emerging Infection Consortium (ISARIC) Global Support Centre, Centre for Tropical Medicine and Global Health, Nuffield Department of Medicine, University of Oxford, Oxford, United Kingdom; 2 Liverpool University Hospitals NHS Foundation Trust, University of Liverpool, Liverpool, United Kingdom; 3 Emory University School of Medicine, Atlanta, Georgia, United States of America; 4 Nuffield Department of Population Health, University of Oxford, Oxford, United Kingdom; 5 Nuffield Department of Population Health, Clinical Trial Service Unit and Epidemiological Studies Unit, University of Oxford, Oxford, United Kingdom; 6 School of Medicine, Brighton and Sussex Medical School, Brighton, United Kingdom; 7 National Institute for Health Research (NIHR) Health Protection Research Unit in Emerging and Zoonotic Infections, Institute of Infection, Veterinary and Ecological Sciences, University of Liverpool, Liverpool, United Kingdom; 8 Walton Centre National Health Service, Foundation Trust, Liverpool, United Kingdom; University Magna Graecia of Catanzaro, ITALY

## Abstract

**Objectives:**

Clinical characterisation studies have been essential in helping inform research, diagnosis and clinical management efforts, particularly early in a pandemic. This systematic review summarises the early literature on clinical characteristics of patients admitted to hospital, and evaluates the quality of evidence produced during the initial stages of the pandemic.

**Methods:**

MEDLINE, EMBASE and Global Health databases were searched for studies published from January 1^st^ 2020 to April 28^th^ 2020. Studies which reported on at least 100 hospitalised patients with Covid-19 of any age were included. Data on clinical characteristics were independently extracted by two review authors. Study design specific critical appraisal tools were used to evaluate included studies: the Newcastle Ottawa scale for cohort and cross sectional studies, Joanna Briggs Institute checklist for case series and the Cochrane collaboration tool for assessing risk of bias in randomised trials.

**Results:**

The search yielded 78 studies presenting data on 77,443 people. Most studies (82%) were conducted in China. No studies included patients from low- and middle-income countries. The overall quality of included studies was low to moderate, and the majority of studies did not include a control group. Fever and cough were the most commonly reported symptoms early in the pandemic. Laboratory and imaging findings were diverse with lymphocytopenia and ground glass opacities the most common findings respectively. Clinical data in children and vulnerable populations were limited.

**Conclusions:**

The early Covid-19 literature had moderate to high risk of bias and presented several methodological issues. Early clinical characterisation studies should aim to include different at-risk populations, including patients in non-hospital settings. Pandemic preparedness requires collection tools to ensure observational studies are methodologically robust and will help produce high-quality data early on in the pandemic to guide clinical practice and public health policy.

**Review registration:**

Available at https://osf.io/mpafn

## Background

The coronavirus (SARS-CoV-2) causing Covid-19 was first notified from Wuhan, China in December 2019. Since then, Covid-19 has spread globally and was declared a pandemic in March 2020 [1]. The response of the global research community has been remarkable, with an exceptional number of Covid-19 publications and unprecedented speed of evidence dissemination.

Covid-19 clinical characterisation studies have been essential in helping to guide clinical decision making and public health policy. These studies may also play a part in characterising the clinical features of new Covid-19 variants. Early in the pandemic, fever, cough and dyspnoea were established as the most common symptoms of Covid-19 [2]. As further clinical studies were conducted, recognition of the wide spectrum of Covid-19 symptoms increased and non-respiratory symptoms such as gastrointestinal [3], cardiovascular [4], and neurological symptoms [5] were reported more frequently. Current case definitions from national and international health bodies such as the World Health Organisation (WHO) and the Centers for Disease Control and Prevention (CDC) include clinical criteria such as diarrhoea, vomiting and headache [6, 7]. Several reviews have summarised the symptoms, biomarkers and radiological findings of Covid-19 studies [8–11] but individual studies have varied in the nature and quality of their evidence at various times during the pandemic. To note, concerns have been raised about the quality of research produced during the pandemic, in time pressured environments and without adequate research infrastructure [12, 13]. Higher quality studies are desirable to guide clinical practice. The aim of this systematic review is to evaluate and assess the quality of the clinical characterisation studies of hospitalised patients produced early in the pandemic to inform research, management and policy making.

## Methods

The research protocol is in line with recommendations outlined in the Preferred Reporting Items for Systematic Reviews and Meta- Analysis (PRISMA) guidelines [14] (S1 Appendix).

### Search strategy

A comprehensive search for studies on Medline (OVID), EMBASE (OVID) and Global Health (OVID) (search date 29 April 2020) was conducted for studies from inception to 28 April 2020. We chose this date limit as we aimed to capture the evidence produced in the early months of the pandemic. Key search terms used were: (Covid-19 OR SARS-CoV-2 OR 2019-nCoV OR novel coronavirus) AND (clinical OR hospital OR admitted) AND (characteristics OR features OR symptoms OR signs), developed with a librarian and piloted prior to use. Studies were restricted to the English language. We did not include pre-prints as these were likely to be updated pending peer reviewed. The electronic database results were supplemented with a Google Scholar search on the 28 April 2020 with the first 100 results screened for inclusion to identify missed peer reviewed articles. Results were uploaded onto EndNote (Clarivate Analytics) and de-duplicated.

### Screening and eligibility

**Fig 1:**
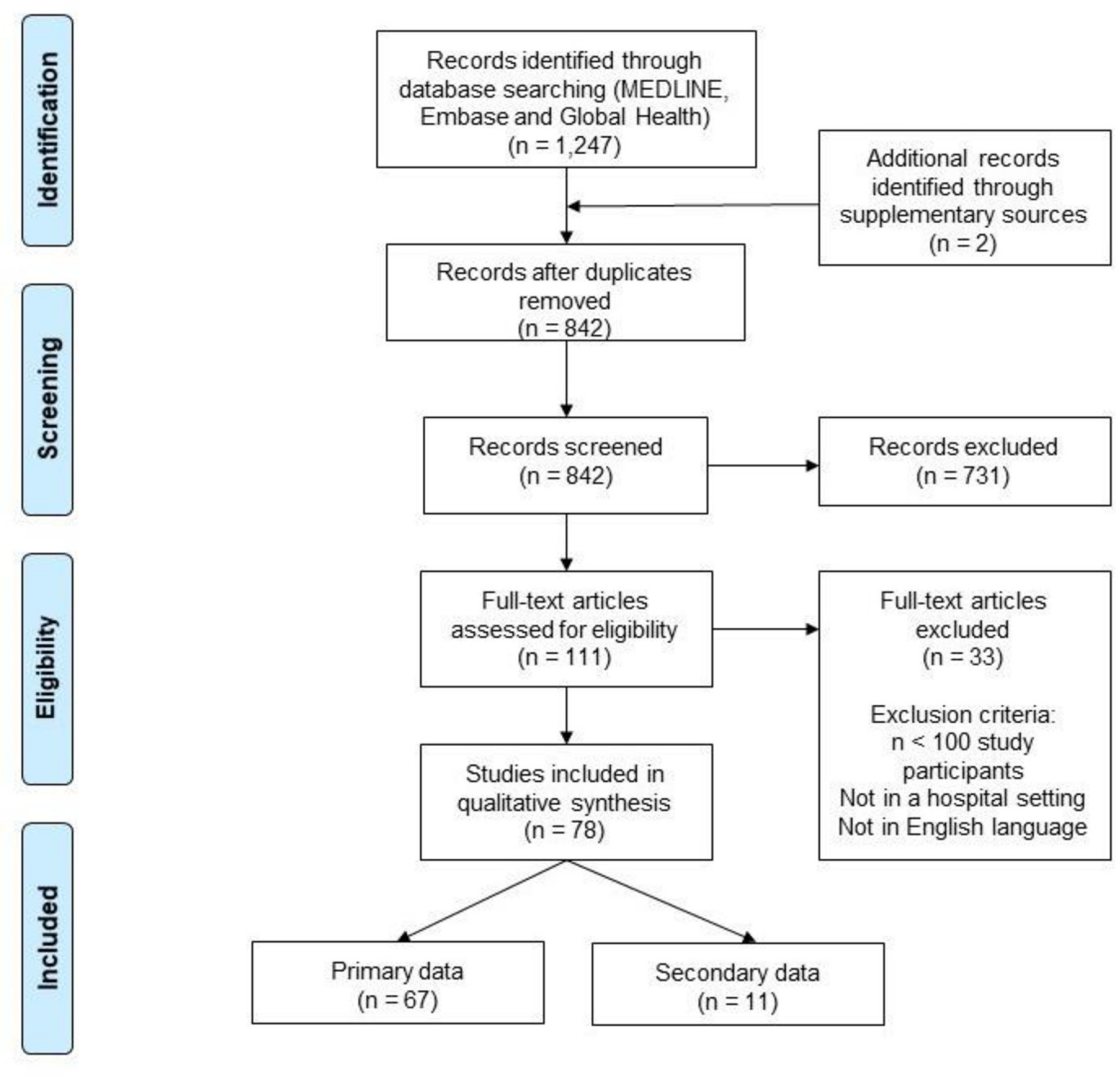
PRISMA diagram for study selection (PRISMA: Preferred Reporting for Systematic Reviews and Meta-Analysis).

Two reviewers independently screened the title and abstract of the retrieved search results. The full text of the articles that passed the first stage for inclusion were divided and screened by two reviewers. Studies presenting clinical data on patients admitted to hospital (ward or ICU) with either clinically or laboratory diagnosed Covid-19 globally were included. We excluded articles which enrolled less than 100 Covid-19 patients to ensure robustness of data and minimise bias (Fig 1).

### Data extraction and synthesis

A standardised form for data extraction was developed and piloted by the reviewers. Data on bibliography, patient demographics, clinical signs and symptoms on admission, biochemical laboratory and imaging results were extracted by one reviewer, with a second reviewer independently checking all the extracted data. The tabulated data were analysed and synthesised by all reviewers.

### Risk of bias assessment

Methodological quality was assessed by two authors, and any discrepancies in scores were resolved though discussion and involvement of a third author if required. The Newcastle Ottawa Scale (NOS) for cohort studies [15] was used to assess the quality of cohort studies with a comparison group, while a modified NOS for cross-sectional studies was used to assess cohort studies without a comparison group and cross sectional studies [16]. Studies identified as case series were evaluated using the Joanna Briggs Institute checklist for case series [17]. Randomised control trials (RCTs) were assessed using the Cochrane risk of bias tool for randomized interventional studies [18].

## Results

**Fig 2:**
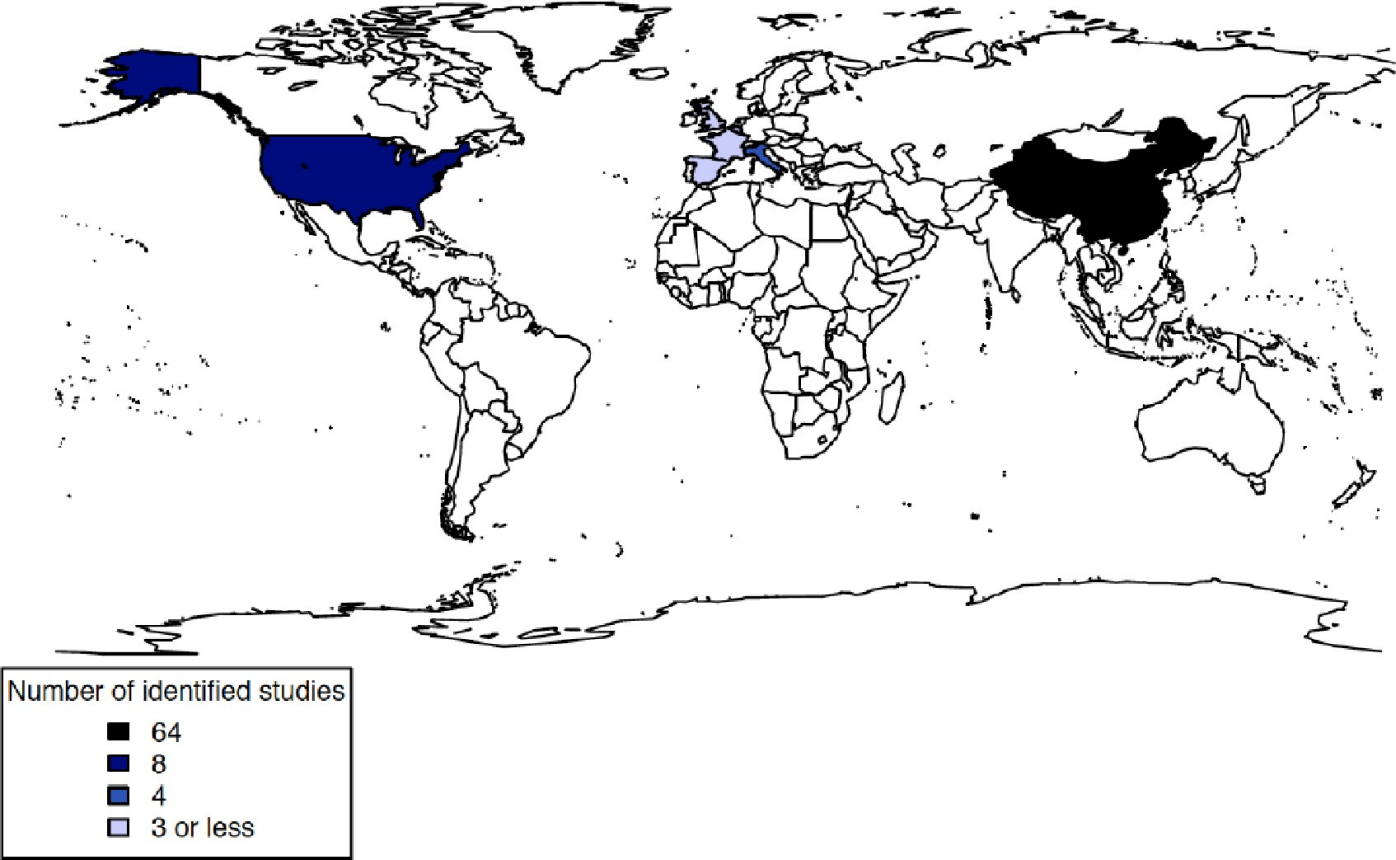
Geographical coverage of the included studies.

The literature search yielded 78 studies that met the inclusion criteria. These studies presented data on 77,483 patients admitted to hospital with Covid-19 in seven countries (Fig 1). There was one RCT, 66 cohort studies, two cross sectional and nine case series studies. The median number of participants was 221 (IQR: 136–424, range: 101–16,749). The majority of studies were set in China (82%), followed by the USA (9%), Italy (4%), France (3%) and the UK (3%). China, the USA and the UK contributed 39%, 36% and 22% of patients included in this article respectively. Two studies were set in more than one country, with one including patients from both the USA and China, and one study including patients from Belgium, France, Italy and Spain (Table 1 and Fig 2). None of the studies identified were set in low or middle income countries (LMICs).

**Table 1:** Number of studies by country and Covid-19 patients included.

Country	Number of studies	Total number of patients
China*	64	30,301
USA*	7	27,705
Italy*	4	2,036
**UK**	2	16,850
France*	3	342
Belgium*	1	104
Spain*	1	104

* Some studies set in more than one country.

The majority of studies sampled populations from both the ward and ICU (87%, n = 67). 3.9% (n = 3) studies included only ICU patients [19–21]. Hospitalised and non-hospitalised patients were sampled in 8% (n = 6) studies [22–27]. Of the included studies, 68% (n = 53) presented clinical data on adults, 1% (n = 1) on children (<18 years), and 23% (n = 18) on both adults and children. The age of participants ranged from <12 months to 96 years old. Data on healthcare workers was presented in 9% (n = 7) studies and 1% (n = 1) studies included pregnant women. Laboratory based methods were specified as the diagnostic method infection in 85% of studies with the remaining studies diagnosing Covid-19 through clinical methods.

A summary table of all included studies is available (S1 Table).

### Risk of bias

The quality scoring systems used for different study designs allowed for assessment of multiple domains including selection of participants, comparability and outcome reporting. Methodological structure and reporting of studies varied in quality. Only 8% (5/66) cohort studies included a comparison group. 36% (n = 24/66) cohort studies controlled for potential confounders such as age or sex, and randomised sampling methods were used in 38% (n = 25) of cohort studies. No cohort studies included sample size calculations. Consecutive enrolment was used in 67% (n = 6/9) case series. Use of laboratory and clinical diagnosis of Covid-19 varied between studies. The RCT had some concerns of bias due to the open label design, which may have affected the outcome measurement of an ordinal scale for clinical improvement. The scores of individual studies are reported in S2–S5 Tables.

### Clinical presentation

The clinical presentation of Covid-19 includes a wide spectrum of disease manifestations. The most commonly reported symptom in studies conducted early in the pandemic was fever, reported in 77% of articles, followed by cough in 71% of articles. These symptoms, both part of the clinical case definition of Covid-19 [28], had a wide prevalence range reported across studies. The highest prevalence of fever (98.6%) was reported in a study of 138 patients with a median age of 56 [29] and the highest prevalence of cough (87%) was reported in a study of 114 patients with a mean age of 47 [27]. The lowest prevalence of both fever (8.9%) and cough (4%) was reported in a study by Lovell et al. of 101 palliative patients with a median age of 81 [30]. Dyspnoea was the third most frequently reported symptom, included in 62% of all studies. The highest reported prevalence of dyspnoea (80%) was in a study of 178 patients, of which 75% were 50 years or older [31]. The lowest reported prevalence of dyspnoea (0.8%) was in a retrospective study of 118 patients with mean age of 44 years [32].

Other commonly reported symptoms early in the pandemic included fatigue, reported in 47% of articles, myalgia in 42%, sore throat in 40%, chest pain in 25%, rhinorrhoea in 22%, and expectoration in 13%. Many patients developed gastrointestinal symptoms including diarrhoea which was reported in 58% of articles, nausea and/or vomiting (40%) and appetite changes (16%). Gastrointestinal symptoms in the absence of respiratory symptoms were reported in 4% of patients on admission in a cohort of 16,749 Covid-19 patients in the UK [33]. Neurological symptoms were also reported in patients, including headache in 44% of articles, altered mental state in 10%, and smell or taste disturbances in 4%. The most commonly reported symptoms and the studies reporting the highest and lowest prevalence of each are reported below (Table 2).

**Table 2:** Prevalence of reported Covid-19 symptoms.

Clinical syndrome	Presenting symptom/sign	N(%)ofallstudies	Lowest prevalence reported				Highest prevalence reported			
			% [^ref^]	Sample size	Age range	Population	% ^ref^	Sample size	Age range	Population
**Respiratory**	Cough (dry/productive)	55 (71.4)	4.0 [30]	101	82(72–89)^+^	Referred to palliative care team	87.0 [27]	114	47(16)*	In- and outpatients with anosmia
	Dyspnoea	48 (62.3)	0.8 [32]	118	44.1(13.6)*	All inpatients	80.0 [31]	178	≥50^ꚙ^	All inpatients
	Sore throat	31 (40.3)	0.7 [39]	298	47(33–61)^+^	All inpatients	43.0 [27]	114	47(16)*	In- and outpatients with anosmia
	Chest pain	19 (24.7)	1.6 [40]	125	38.8(13.8)*	All inpatients	65.2 [41]	112	65(49–70.8)^+^	All inpatients
	Expectoration	19 (13.0)	4.4 [42]	137	57^+^	Admitted to respiratory ward	34.9 [43]	645	46.7(13.8)*	All inpatients
	Rhinorrhoea	17 (22.1)	1.0 [39]	298	47(33–61)^+^	All inpatients	35.3 [44]	136	69(61–77)^+^	Inpatients post in hospital cardiac arrest
**Gastrointestinal**	Diarrhoea	45 (58.4)	1.0 [45]	131	47(15)*	All inpatients	52.0 [27]	114	47(16)*	In- and outpatients with anosmia
	Nausea and/or vomiting	31 (40.3)	0.5 [46]	889	47.8(15.2)*	All inpatients	35.0 [27]	114	47(16)*	In- and outpatients with anosmia
	Changes in appetite and or /anorexia	12 (15.6)	3.0 [47]	120	45.4(15.6)*	All inpatients	78.6 [48]	204	52.9(16)*	All inpatients
**Neurological**	Headache	34 (44.2)	0.9 [49]	108	52(37–58)^+^	All inpatients	82.0 [27]	114	47(16)*	In- and outpatients with anosmia
	Altered mental state (confusion/agitation)	8 (10.4)	1.4 [50]	1590	44.2(14.8)*	All inpatients	42.6 [30]	101	82(72–89)^+^	Referred to palliative care team
	Smell or taste impairment	3 (3.9)	5.6 [34]	214	52.7(15.5)*	All inpatients	88.8 [26]	417	36.9(11.4)^+^	Ward and outpatients
**Systemic**	Fever	59 (76.6)	8.9 [30]	101	82(72–89)^+^	Referred to palliative care team	98.6 [29]	138	56(42–69)^+^	All inpatients
	Myalgia and/or arthralgia	32 (41.6)	2.0 [45]	131	47(15)*	All inpatients	74.0 [27]	114	47(16)*	In- and outpatients with anosmia
	Fatigue	36 (46.8)	4.7 [39]	298	47(33–61)^+^	All inpatients	93.0 [27]	114	47(16)*	In- and outpatients with anosmia

^+^Median (IQR)

*Mean (SD)

^ꚙ^75% of patients aged ≥ 50 ‘All inpatients’ included patients with mild, moderate and severe disease.

In addition, Covid-19 complications and their associated symptoms were found to sometimes be presenting features of the disease. In a study of 214 Covid-19 infected patients, acute cerebrovascular events affected 2.8% of included participants, with two of these patients presenting to the Emergency Department with neurological symptoms in the absence of respiratory symptoms [34], Five of the included studies reported data on Covid-19 incubation period, with the minimum and maximum incubation period reported in studies ranging from 3 to 6.7 days [22, 35–38].

### Laboratory findings

Laboratory results were reported in 54 studies. Although most studies reported laboratory values on admission, early Covid-19 studies often did not define collection timepoint. Lymphocytopenia was the most common laboratory finding among patients admitted to hospital with confirmed Covid-19, being reported in 32% (n = 25/78) of studies [35, 43, 51–56]. The highest prevalence of lymphocytopenia (99.1%) was reported in a study of 225 patients with a mean age of 50 years [57]. The second most commonly reported laboratory finding was elevated C-reactive protein (CRP) in 19% (n = 15) of studies.

Other abnormal laboratory findings included thrombocytopenia, elevated erythrocyte sedimentation rate (ESR), lactate dehydrogenase (LDH), D-dimer, troponin and cytokine levels, particularly interleukin 6 (IL-6). Aspartate aminotransferase (AST) and alanine aminotransferase (ALT) were both elevated, and decreased albumin and leukopenia were reported in several studies [35, 43, 51–56, 58–61] (Table 3).

**Table 3:** Abnormal laboratory findings in Covid-19.

Laboratory finding	N (%) of total studies	Lowest prevalence reported				Highest prevalence reported			
		% ^ref^	Lab cut-off level	Sample size	Age median/mean (variation)	% ^ref^	Lab-cut off level	Sample size	Age median/mean (variation)
Lymphocytopenia	25 (32)	26.1 [64]	<0.8*10^^9^/L	161	45 (33.5–57)^+^	99.1 [57]	Not defined	225	50 (14)*
Elevated CRP	15 (19)	55 [65]	>6.0 mg/L	149	62 (44–70)^+^	91.9 [66]^ ^	>3.0 mg/L	136	57^+^
Leukopenia	14 (18)	8 [45]	<3.5*10^^9^/L	131	47 (15)*	41 [64]	<4.0*10^^9^/L	161	45 (33.5–57)^+^
Elevated ALT	12 (15)	8.1 [64]	>40 u/L	161	45 (33.5–57)^+^	39 [54]^ ^	>60 u/L	2176	63 (52–75)^+^
Leukocytosis	12 (15)	1.3 [67]	Not defined	150	56^+^	23.4 [68]^ ^	>9.5*10^^9^/L	197	51 (43–60)^+^
Elevated AST	11 (14)	5.7 [69]	>40 u/L	417	47 (33–59)^+^	58 [54]^ ^	>40 u/L	3263	63 (52–75)^+^
Elevated D-dimer	11 (14)	14 [65]	>0.55 mg/L	149	45.1 (13.4)*	77.5 [61]^ ^	>0.5 mg/L	661	63 (50–71)^+^
Thrombocytopenia	10 (13)	7 [70]	<100*10^^9^/L	191	56 (46–67)^+^	36.2 [24]	<150*10^^9^/L	869	47 (35–58)^+^
Elevated LDH	10 (13)	23.6 [64]	>225 u/L	161	45 (33.5–57)^+^	75 [40]	>250 u/L	125	41.5 (15.1)*
Elevated procalcitonin	9 (12)	2.4 [40]	>0.5 ng/mL	125	41.5 (15.1)*	70 [71]	>0.5 ng/mL	236	62 (44–70)^+^
Elevated ESR	7 (9)	62.7 [40]	>15 mm/h	125	41.5 (15.1)*	93.8 [68]^ ^	>15 mm/h	194	51 (43–60)^+^
Elevated troponin	5 (6)	12.7 [38]	>26 pg/mL	55	54 (37–67)^+^	41 [71]	>15.6 pg/mL	274	62 (44–70)^+^

+ Median (IQR)

*Mean (SD) Studies included in this table are those that reported on lab value prevalence.

Abbreviations: CRP: C-reactive protein ALT: alanine aminotransferase AST: Aspartate aminotransferase ESR: Erythrocyte sedimentation rate LDH: lactate dehydrogenase.

In studies which compared laboratory values among mild and severe cases of Covid-19, those with severe disease were found to have more prominent lab abnormalities including lower lymphocyte counts, higher inflammatory marker levels, (CRP, ESR, LDH), and elevated D-dimer levels and liver enzymes (AST, ALT) [24, 39, 52, 55, 56, 62, 63]. Among studies that reported immune markers, higher levels of serum cytokines and lower levels of T lymphocytes were associated with disease severity [56, 62].

### Imaging findings

Imaging findings were reported in 44 studies. Studies from early in the pandemic demonstrated that imaging findings may be normal in early or mild disease. In a study of 298 laboratory confirmed patients, 14.8% (44/298) of all patients had a normal chest computed tomography (CT) scan on admission [39]. CT imaging was more likely to be normal the sooner it was conducted after symptom onset [39]. In a study of 121 laboratory confirmed patients, 56% (n = 20/36) of patients had a normal CT scan 0 to 2 days post-symptom onset [72], while a study of 112 patients reported a normal scan in 21% (n = 10/47) of patients 0 to 4 days post-symptom onset [73]. A study of 543 patients admitted to a Chinese hospital found that the median time from symptom onset to the diagnosis of pneumonia on CT was 4 days [56]. Disease progression on repeated imaging was reported in a small number of studies. In a study of 248 confirmed cases who had repeated scans, 66% (n = 163/248) showed disease progression after a median of 3 days [74], while in a study of 149 patients where 17 had initial normal radiological findings, 29% (5/17) had disease progression on imaging after a median of 7 days [65].

Common abnormalities seen on computed tomography (CT) at admission included ground glass opacities and consolidation (Table 4) [29, 52, 55, 57, 70, 71, 75]. Reported prevalence of ground glass opacities ranged from 12.1% to 96% in the included studies [52, 65]. Other frequently reported Covid-19 CT imaging features include a peripheral distribution of lesions [32, 47, 65, 72, 76], and multi-lobar involvement [45, 56, 77, 78]. Less common CT abnormalities included pleural effusion and lymphadenopathy [47, 52, 79].

**Table 4:** Imaging findings in Covid-19.

Imaging findings	N (%) of all studies^ⴕ^	Lowest prevalence documented				Highest prevalence documented			
		%^ref^	Sample size	Age Mean (SD)	Population details	%^ref^	Sample size	Age +median (IQR) mean (SD)	Population details
Bilateral infiltrates	26(33)	43.6 [63]	280	43.1(19.0)	All inpatients	100 [75]	135	47(36–55)^+^	All inpatients
Ground glass opacities	25 (32)	12.1 [65]	149	45.1(13.4)	All inpatients	96.2 [52]	476	53(40–64)^+^	All inpatients
Consolidation	12 (15)	7.2 [65]	149	45.1(13.4)	All inpatients	59 [70]	191	56(46–67)^+^	All inpatients
Lower lobe infiltrates	7 (9)	42.4 [32]	118	44.1(13.6)	All inpatients	95 [78]	234	44.6(14.8)*	All inpatients
Multilobar disease	12 (15)	35.7 [43]	645	46.7(13.8)	All inpatients	94.6 [56]	548	60(48–69)^+^	All inpatients
Peripheral distribution	9 (12)	35.9 [65]	149	45,1(13.4)*	All inpatients	91 [72]	121	45.3(15)*	All inpatients with a CT scan

^ⴕ^Includes studies with both CT and X-ray findings. ‘All inpatients’ included patients with mild, moderate and severe disease

## Discussion

In this systematic review we evaluated Covid-19 studies presenting data on clinical characteristics of hospitalised patients published in the early months of the pandemic in the first quarter of 2020. Most of the studies identified were observational cohort studies with limited control studies, set in China, the USA and in Europe. Commonly reported symptoms in hospitalised patients early in the pandemic were fever and cough. Laboratory and imaging findings were diverse, with lymphocytopenia and ground glass opacities the most frequently reported findings, respectively. The studies were heterogenous, limiting comparability.

Observational studies are useful in providing estimates of disease characteristics and outcomes in real-world populations and help to generate hypotheses which can be explored in further research. These studies had an important role in helping define criteria for clinical diagnosis of Covid-19 [6, 7, 80], guiding clinical practice and provided early indicators of future research priorities–nevertheless, when observational evidence is generated at such an early stage, and with evidence acquired soon thereafter, these clinical criteria may be restrictive and would need to be revisited and updated [81]. This type of study design can also be carried out relatively quickly especially if pre-positioned data collection tools are available to ensure methodological robustness and consistency.

A limitation of observational studies is that they are subject to numerous sources of bias [82]. Most of the included clinical characterisation studies had a moderate to high risk of bias. This bias may have contributed to the wide range of clinical characteristic prevalence values which were reported. We found that over half of the studies did not control for potential confounding factors such as age or sex and only 8% of studies included a comparison arm of Covid-19 negative patients. This means being able to establish an association between a symptom and Covid-19, but not the predictive value of the presence or absence of a symptom for Covid-19. Many studies did not report the length of illness or the time point at which laboratory tests were taken, which would aid in understanding the temporal course of disease. These methodological factors should be considered when designing observational studies, especially early in a pandemic, to ensure high quality evidence is generated. While it may not be possible to identify a control group, it is nonetheless important to standardise data collection tools and analyses. The International Severe Acute Respiratory and emerging Infections Consortium (ISARIC) WHO Clinical Characterisation protocol study [83] is an example of a methodologically rigorous study aiming to characterise clinical features and risk factors of patients admitted to hospital with Covid-19. This prospective observational multi-site cohort study uses a pre-prepared suite of protocols and agreements allowing it to commence early in the pandemic and rapidly enrol a large sample size. The first report from this study, published in April 2020, had the largest sample of patients of studies included in this review with 16,749 patients from the UK [33]. This study was one of the first reports of Covid-19 patients presenting solely with gastrointestinal symptoms, highlighting the importance of large sample sizes to capture less frequent clinical characteristics. The data from this study was further developed into a risk score to predict mortality in patients hospitalised with Covid-19 [84, 85], again underlining the importance of data from well-designed observational studies to inform clinical management. Meanwhile data and knowledge have accumulated: for comparison, the latest update of the report [86] has 305,241 patients from 64 countries.

The generalisability of these results to other population with different demographics, risk factors and healthcare systems needs to be considered. Over 85% of the studies were set in China, which may limit the applicability of any inferences from these results to different demographics. Subsequent data from later in the pandemic has highlighted worse outcomes of Covid-19 infection in Black, Asian and minority ethnic groups in certain settings [87]. We found no studies reporting patient cohorts from LMICs despite both Egypt and Brazil reporting initial Covid-19 cases, as early as February 2020 [88, 89]. This is likely due to the under-recognition and lack of testing capabilities in the early stages of the pandemic, and demonstrates how the timescale of pandemic development, geographical origins and demographics of the first affected countries can influence early clinical characterisation of the disease [90]. Data sharing between high income countries (HIC) and LMICs with appropriate data governance in place will be an important tool in the global research response, however assessing clinical characteristics in low resource settings is crucial given different population age structure and risk factor profiles compared to HIC populations. For example, the prevalence of respiratory disease risk factors is higher in all age groups in LMICs compared to HICs and pneumonia is the leading cause of death in children in LMICs [91]. This different risk factor profile could mean varied clinical characteristics and outcomes compared to HICs, emphasising the need for clinical studies in settings with different demographic, epidemiology, and income patterns.

Some studies included patients of all ages inclusive of children, however the number of children enrolled in these studies was low and wide age brackets were used in the reporting of results. This paucity of data likely reflects the lower hospitalisation rates for children with Covid-19 [92]. However, inclusion of children in early clinical studies is important to ensure the range of characteristics is captured as early as possible, helping to guide clinical decision making in paediatric patients. Subsequent studies have demonstrated that severe disease in children is rare however more likely in children with underlying co-morbidities [92], and a severe multisystem inflammatory syndrome has been reported [93, 94].

Limitations of this review included our focus on only hospitalised patients due to the limited data available from primary care and community settings in the early stages of the pandemic. Hospitalised patients are likely to represent the more severe end of the clinical spectrum, presenting with a more advanced clinical picture compared to cases in the community. We included studies with at least 100 patients to ensure robustness, however this meant we did not assess smaller cohorts or case reports. We included both clinically and laboratory diagnosed Covid-19 which may have reduced accuracy of diagnosis. Furthermore, evaluating the progression of clinical characteristics was challenging as many studies did not report the day since symptom onset on which results were recorded. Some articles may have been missed due to limiting the inclusion of studies to those published in the English language.

## Conclusion

This review reflects our knowledge of clinical characteristics of Covid-19 in the earlier months of the pandemic, and highlights the context in which early clinical and public health decisions were made. The early Covid-19 literature had a moderate to high risk of bias and presented several methodological issues. While these studies were useful in informing clinical and public health decisions in the early stages of the pandemic, clinicians should adopt a cautious approach when using evidence from the early literature. These data highlight the need for studies conducted early in an epidemic to include different at risk populations, including patients with different degrees of severity and cases from non-hospital settings. Although research conducted in the initial stages of an outbreak is often time pressured, pandemic preparedness means having standardised collection tools right at the beginning of the pandemic to ensure observational studies are methodologically robust, and will help produce high-quality data to guide clinical practice and public health policy.

## Supporting information

S1 Appendix PRISMA systematic review checklist. (DOC)

S1 Table Individual study details. (XLSX)

S2 Table Risk of bias assessment scores. The Newcastle-Ottawa Scale for cross-sectional studies was adapted for cohort studies without a comparison group and cross sectional studies and used to assess the risk of bias for each included study [1].
(DOCX)

S3 Table Risk of bias assessment scores for cohort studies with a comparison group. The Newcastle-Ottawa Scale for cohort studies was used to assess the risk of bias for each included study [1].
(DOCX)

S4 Table Risk of bias assessment scores for Case series studies. The Joanna Briggs Institute Checklist for Case Series was used to assess the risk of bias for each included study [1].
(DOCX)

S5 Table Risk of bias assessment scores for RCT studies. The Cochrane risk-of-bias tool for randomised trials was used to assess the risk of bias for included RCTs. [1].
(DOCX)
