# Supplementary material for: Evaluating clinical characteristics studies produced early in the Covid-19 pandemic: A systematic review
Source: PLoS One. 2021 May 18;16(5):e0251250. doi: 10.1371/journal.pone.0251250 (PMC8130955; doi:10.1371/journal.pone.0251250)
Supplement: S2 Table — The Newcastle-Ottawa Scale for cross-sectional studies was adapted for cohort studies without a comparison group and cross sectional studies and used to assess the risk of bias for each included study [1]. (DOCX) [file pone.0251250.s003.docx]

| Study | Selection | | | Comparability | Outcome | | Total score /9 |
| --- | --- | --- | --- | --- | --- | --- | --- |
|  | Representativeness of the sample | Sample size | Ascertainment of the exposure | Comparability of subjects in different outcome groups | Assessment of outcome | Statistical test |  |
| Docherty et al | ** | - | ** | * | ** | * | 8 |
| CDC COVID-19 Response Team | * | - | ** | - | * | - | 4 |
| Zhang et al | * | - | ** | * | * | * | 6 |
| Liang et al | * | - | ** | * | ** | * | 7 |
| Guan et al | * | - | ** | - | ** | * | 6 |
| Ji et al | * | - | ** | - | * | * | 5 |
| Wang et al | * | - | ** | - | ** | * | 6 |
| Wang et al | * | - | * | - | * | * | 4 |
| Lian et al | * | - | ** | - | * | * | 5 |
| Cheng et al | * | - | ** | * | ** | * | 7 |
| Zhang et al | * | - | ** | * | ** | * | 7 |
| Zhang et al | * | - | * | * | * | * | 5 |
| Li et al | * | - | ** | * | * | * | 6 |
| Feng et al | ** | - | ** | * | ** | * | 8 |
| Qin et al | ** | - | ** | - | ** | * | 7 |
| Lechien et al | * | - | ** | - | * | * | 5 |
| Cai et al | * | - | ** | - | * | * | 5 |
| Shi et al | ** | - | ** | * | ** | * | 8 |
| Myers et al | * | - | ** | - | ** | * | 6 |
| Wang et al | ** | - | ** | * | ** | * | 8 |
| Lu et al | ** | - | ** | - | ** | - | 6 |
| Cai et al | ** | - | ** | * | ** | * | 8 |
| Wu et al | * | - | ** | * | ** | * | 7 |
| Chen et al | * | - | ** | - | ** | * | 6 |
| Tian et al | * | - | ** | - | * | * | 5 |
| Chen et al | * | - | ** | * | * | * | 6 |
| Liu et al | * | - | ** | * | ** | * | 7 |
| Colombi et al | * | - | ** | * | ** | * | 7 |
| Dai et al | * | - | ** | - | * | * | 5 |
| Li et al | * | - | ** | - | * | * | 5 |
| Yan et al | * | - | ** | * | ** | * | 7 |
| Zhang et al | * | - | ** | - | ** | * | 6 |
| Han et al | * | - | ** | - | * | * | 5 |
| Pan et al | * | - | ** | - | ** | * | 6 |
| Wu et al | * | - | ** | - | ** | * | 6 |
| Zhou et al | ** | - | ** | * | ** | * | 8 |
| Shi et al | * | - | ** | - | * | * | 5 |
| Garg et al. | * | - | ** | - | * | * | 5 |
| Guo et al | * | - | ** | - | * | * | 5 |
| Zheng et al | * | - | * | - | * | * | 4 |
| Mo et al | ** | - | ** | * | * | * | 7 |
| Fan et al | * | - | ** | * | * | * | 6 |
| Yang et al | * | - | ** | - | ** | * | 6 |
| Fan et al | * | - | ** | - | * | * | 5 |
| Zhang et al | ** | - | ** | - | - | * | 6 |
| Hu et al | * | - | * | - | ** | * | 6 |
| Liu et al | * | - | ** | - | ** | * | 6 |
| Shao et al | * | - | * | * | ** | * | 6 |
| Cao et al | ** | - | * | - | - | * | 4 |
| Wan et al | * | - | ** | - | ** | * | 6 |
| Bernheim et al | * | - | ** | - | * | - | 4 |
| Zhang et al | ** | - | ** | - | * | * | 6 |
| Chen et al | * | - | * | - | ** | - | 4 |
| Zhao et al | * | - | ** | - | * | * | 5 |
| Wang et al | * | - | ** | - | ** | * | 6 |
| Klopfenstein et al | ** | - | ** | - | * | * | 6 |
| Wang et al | * | - | * | - | * | * | 4 |
| Ding et al | * | - | ** | - | * | * | 5 |
| Deng et al | ** | - | ** | * | ** | * | 8 |
| Yao et al | ** | - | ** | * | ** | * | 8 |
| Cao et al | ** | - | ** | - | ** | * | 7 |
| Zhao et al | * | - | ** | - | * | * | 5 |

**S2 Table: Risk of bias assessment scores. The Newcastle-Ottawa Scale for cross-sectional studies was adapted for cohort studies without a comparison group and cross sectional studies and used to assess the risk of bias for each included study [1].**

1. Wells GA, Shea B, O’Connell Da, Peterson J, Welch V, Losos M, et al. The Newcastle-Ottawa Scale (NOS) for assessing the quality of nonrandomised studies in meta-analyses: Oxford; 2000 [Available from: <http://www.ohri.ca/programs/clinical_epidemiology/oxford.asp>.
