# Supplementary material for: Evaluating clinical characteristics studies produced early in the Covid-19 pandemic: A systematic review
Source: PLoS One. 2021 May 18;16(5):e0251250. doi: 10.1371/journal.pone.0251250 (PMC8130955; doi:10.1371/journal.pone.0251250)
Supplement: S3 Table — The Newcastle-Ottawa Scale for cohort studies was used to assess the risk of bias for each included study [1]. (DOCX) [file pone.0251250.s004.docx]

| Study | Selection | | | | Comparability | Outcome | | | Total score /9 |
| --- | --- | --- | --- | --- | --- | --- | --- | --- | --- |
|  | Representativeness of the exposed cohort | Selection of the non-exposed cohort | Ascertainment of the exposure | Outcome of interest was not present at start of study | Comparability of cohorts | Assessment of outcome | Was follow-up long enough for outcomes to occur | Adequacy of follow-up of cohorts |  |
| Ferrari et al | ** | * | ** | - | - | * | - | * | 7 |
| Simonnet et al | ** | * | ** | - | * | * | * | - | 8 |
| Zhang et al | * | * | ** | - | - | * | * | - | 6 |
| Bai et al | * | - | ** | - | - | - | * | - | 4 |
| Yin et al | * | * | ** | - | - | * | * | - | 6 |

**S3 Table: Risk of bias assessment scores for cohort studies with a comparison group. The Newcastle-Ottawa Scale for cohort studies was used to assess the risk of bias for each included study [1]**

1. Wells GA, Shea B, O’Connell Da, Peterson J, Welch V, Losos M, et al. The Newcastle-Ottawa Scale (NOS) for assessing the quality of nonrandomised studies in meta-analyses: Oxford; 2000 [Available from: <http://www.ohri.ca/programs/clinical_epidemiology/oxford.asp>.
