# Supplementary material for: Evaluating clinical characteristics studies produced early in the Covid-19 pandemic: A systematic review
Source: PLoS One. 2021 May 18;16(5):e0251250. doi: 10.1371/journal.pone.0251250 (PMC8130955; doi:10.1371/journal.pone.0251250)
Supplement: S4 Table — The Joanna Briggs Institute Checklist for Case Series was used to assess the risk of bias for each included study [1]. (DOCX) [file pone.0251250.s005.docx]

| Study | Were there clear criteria for inclusion? | Was the condition measured in a standard, reliable way for all participants? | Were valid methods used for identification of the condition? | Did the case series have consecutive inclusion of participants? | Did the case series have complete inclusion of participants? | Was there clear reporting of participant demographics? | Was there clear reporting of clinical information? | Were the outcomes or follow up results clearly reported? | Was there clear reporting of the presenting site(s)/clinic(s) demographic information? | Was statistical analysis appropriate? |
| --- | --- | --- | --- | --- | --- | --- | --- | --- | --- | --- |
| Richardson et al | Y | Y | Y | Y | Y | Y | Y | Y | Y | Y |
| Dong et al | Y | Y | Y | Unclear | Unclear | N | N | Unclear | Unclear | Y |
| Grasselli et al | Y | Y | Y | Y | Y | N | Y | Y | Y | Y |
| Goyal et al | Y | Y | Y | Y | Unclear | Y | Y | Y | Y | Y |
| Mao et al | Y | Y | Y | Y | Y | Y | Y | Y | N | Y |
| Wang et al | Y | Y | Y | Y | Unclear | Y | Y | Y | Y | Y |
| Wang et al | Y | Y | Y | Unclear | Unclear | Y | Y | Y | Y | Y |
| Li et al | Y | Y | Y | Unclear | Unclear | N | N | Y | Y | Y |

**S4 Table: Risk of bias assessment scores for Case series studies. The Joanna Briggs Institute Checklist for Case Series was used to assess the risk of bias for each included study [1]**

1. Munn Z, Barker T, Moola S, Tufanaru C, Stern C, A. M, et al. Methodological quality of case series studies, JBI Evidence Synthesis. 2020.
