# Supplementary material for: Evaluating clinical characteristics studies produced early in the Covid-19 pandemic: A systematic review
Source: PLoS One. 2021 May 18;16(5):e0251250. doi: 10.1371/journal.pone.0251250 (PMC8130955; doi:10.1371/journal.pone.0251250)
Supplement: S5 Table — The Cochrane risk-of-bias tool for randomised trials was used to assess the risk of bias for included RCTs. [1]. (DOCX) [file pone.0251250.s006.docx]

| **Study** | **Risk of bias from randomisation process** | **Risk of bias due to deviations from intended interventions** | **Missing outcome data** | **Risk of bias in measurement of the outcome** | **Risk of bias in selection of reported result** | **Overall bias** |
| --- | --- | --- | --- | --- | --- | --- |
| Cao et al. | Low | Some concerns | Low | Low | Some concerns | Some concerns |

**S5 Table: Risk of bias assessment scores for RCT studies. The Cochrane risk-of-bias tool for randomised trials was used to assess the risk of bias for included RCTs. [1]**

1. Higgins JPT, Altman DG, Gøtzsche PC, Jüni P, Moher D, Oxman AD, et al. The Cochrane Collaboration’s tool for assessing risk of bias in randomised trials. BMJ. 2011;343:d5928.
